# Variations in household microclimate affect outdoor-biting behaviour of malaria vectors

**DOI:** 10.12688/wellcomeopenres.12928.1

**Published:** 2017-10-24

**Authors:** Halfan S. Ngowo, Emmanuel Wilson Kaindoa, Jason Matthiopoulos, Heather M. Ferguson, Fredros O. Okumu

**Affiliations:** 1 Department of Environmental Health and Ecological Sciences, Ifakara Health Institute, Ifakara, Tanzania; 2 Institute of Biodiversity, Animal Health and Comparative Medicine, University of Glasgow, Glasgow, UK; 3 School of Public Health, University of the Witwatersrand, Parktown, South Africa

**Keywords:** Anopheles, GAMM, GLMM, indoor biting, malaria, microclimate, outdoor biting, rainfall

## Abstract

**Background**: Mosquito behaviours including the degree to which they bite inside houses or outside is a crucial determinant of human exposure to malaria. Whilst seasonality in mosquito vector abundance is well documented, much less is known about the impact of climate on mosquito behaviour. We investigated how variations in household microclimate affect outdoor-biting by malaria vectors, *Anopheles arabiensis *and *Anopheles funestus*.

**Methods**: Mosquitoes were sampled indoors and outdoors weekly using human landing catches at eight households in four villages in south-eastern Tanzania, resulting in 616 trap-nights over 12 months. Daily temperature, relative humidity and rainfall were recorded. Generalized additive mixed models (GAMMs) were used to test associations between mosquito abundance and the microclimatic conditions. Generalized linear mixed models (GLMMs) were used to investigate the influence of microclimatic conditions on the tendency of vectors to bite outdoors (proportion of outdoor biting).

**Results**: *An. arabiensis* abundance peaked during high rainfall months (February-May), whilst *An. funestus *density remained stable into the dry season (May-August) *.* Across the range of observed household temperatures, a rise of 1 ^º^C marginally increased nightly *An. arabiensis* abundance (~11%), but more prominently increased *An. funestus *abundance (~66%). The abundance of *An. arabiensis *and *An. funestus* showed strong positive associations with time-lagged rainfall (2-3 and 3-4 weeks before sampling). The degree of outdoor biting in *An. arabiensis* was significantly associated with the relative temperature difference between indoor and outdoor environments, with exophily increasing as temperature inside houses became relatively warmer. The exophily of *An. funestus* did not vary with temperature differences.

**Conclusions**: This study demonstrates that malaria vector *An. arabiensis *shifts the location of its biting from indoors to outdoors in association with relative differences in microclimatic conditions. These environmental impacts could give rise to seasonal variation in mosquito biting behaviour and degree of protection provided by indoor-based vector control strategies.

## Introduction

Malaria control is entering a crucial stage in sub-Saharan Africa, with significant investments and gains being made ^1^. While the disease still kills 429,000 people annually ^1^, the scale-up of key interventions such as Long-Lasting Insecticide Nets (LLINs), indoor residual spraying (IRS) and treatment with artemisinin combination drugs are estimated to have reduced malaria incidence and mortality by 21% and 29% respectively between 2010 and 2015 ^1, 2^. Despite their considerable impact, LLINs and IRS cannot provide complete malaria suppression on their own ^3, 4^, partly because they target mainly indoor biting and indoor resting mosquitoes ^4, 5^. In many persistent malaria transmission settings, a considerable amount of transmission is potentially maintained by malaria vectors that predominantly bite outdoors ^6^, or are physiologically resistant to the insecticides used for LLINs and IRS ^7^. For example *Anopheles arabiensis* and *Anopheles funestus* have been observed to bite early in the evening or early morning when people are outdoors and thus unprotected by LLINs or IRS ^8– 11^. Targeting these vectors of persistent transmission is one of the next steps towards malaria elimination.

While vector species are often described as having relatively fixed patterns of behaviour, there are indications that vectors may shift their biting behaviour in response to environmental conditions ^12, 13^ and to avoid contact with insecticides used indoors ^9, 14^. Although there is recognition that mosquitoes are capable of adapting their host choice and resting behaviours ^15– 17^, there is limited understanding of the role of the fine-scale household-level climatic conditions in determining the timing and location of vector biting. For example, it has been widely demonstrated that mosquito vector abundance varies significantly in response to seasonal changes in climate and rainfall ^18– 21^, but much less is known about whether there are corresponding seasonal changes in the specific timing and location of their biting, or even choice of resting habitats. Given the crucial importance of outdoor biting as a determinant of the degree of protection that can be provided by LLINs, it is crucial to understand if and how this vector behaviour may vary in response to microclimatic variation. Such information is critical for predicting and quantifying human exposure to mosquito bites throughout the year, and assessment of the degree of biological coverage that can be achieved with particular interventions.

Environmental conditions influence mosquito vector life-history and demography in several ways. Firstly as mosquitoes are ectotherms, their development and survival is dependent on the temperature of surrounding environments ^22, 23^. Temperature and humidity have strong impacts on the rate of mosquito and parasite development, larval development rates and mosquito biting rates which in turn determines malaria transmission intensity ^22– 28^. In tropical areas, malaria vectors are exposed to extensive environmental variation throughout the annual seasonal cycle of rainfall, which is characterized by periods of high rainfall and cooler temperatures, followed by dry periods where temperatures are hotter. This variability causes high amplitude fluctuations in mosquito abundance ^29, 30^ and corresponding malaria transmission ^31, 32^. In addition to the impacts of temperature and humidity described above, rainfall has a significant independent impact on mosquito abundance through its role in creating aquatic habitats for larval development ^21, 33– 36^.

In addition to rainfall, seasonal variation in temperature can have numerous impacts on mosquito demography and transmission potential. For example, the time required for *An. gambiae s.l.* to develop from egg to pupa is highly dependent on temperature, lasting from 9.3 days at 35°C, and increasing to 12.6 days at 25°C ^37^. The duration of the mosquito gonotrophic period (time between blood-feeding and egg-laying) is also temperature-dependent ^27^. High ambient temperature (e.g.> 32°C) results in a faster rate of blood meal digestion, thus shorter period between feeding cycles, and higher overall biting frequencies ^35^. These increases in mosquito development and life history are expected to increase with temperature up to a maximum threshold, above which temperature becomes lethal for mosquitoes. Also, the extrinsic incubation period (EIP) of malaria parasites developing within mosquitoes depends on temperature ^28^. The sporogonic cycle of *Plasmodium falciparum* requires a minimum temperature of 16°C, below which parasite development will not be completed. The duration of EIP is reduced with increasing temperature ^38^ until a certain threshold, beyond which mosquito and parasites die before the cycle is complete ^26, 39^.

Whilst the effects of seasonal climatic variation on mosquito and parasite development are relatively well known, much less is understood about its impact on mosquito biting behaviour and associated human exposure. For example, the tendency of vectors to bite and rest indoors versus outside is a key determinant of how much protection can be obtained through use of LLINs or IRS ^40^. The relative degree of preference for biting indoors (endophily) is often assumed to be fixed within a vector species, with African vectors such as *An. gambiae* and *An. funestus* often described as being near exclusively endophilic ^41– 43^. However, other more behaviourally plastic species such as *An. arabiensis* can bite both indoors and outdoors ^9^.

The relative contribution of genetic versus environmental factors to the observed heterogeneity in these and other mosquito behaviours is poorly understood ^44, 45^. It is possible that the degree of endophily in a vector population is influenced by relative differences in temperatures and humidity of indoor and outdoor locations. For example, vectors may switch their activity between an indoor and outdoor environment depending on which is most optimal for their fitness ^46^. Some studies have investigated the effect of indoor temperature and humidity on mosquito abundance ^23, 46, 47^, but to our knowledge none have tested for association with indoor vs. outdoor biting activity. In rural Africa, indoor microclimates vary greatly due to variables such as house density, building design, construction materials and seasonal variation in climate ^48^.

Although vectors are known to be capable of adjusting their biting and resting habitats in response to climate under both laboratory ^49^ and field settings ^50, 51^, little is known about whether seasonal variation in microclimatic conditions (temperature and humidity) is sufficient to alter their biting behaviour around human dwellings. If so, this could give rise to seasonal variation in the degree of coverage provided by vector control measures such as LLINs. The main aim of this study was therefore to quantify the fine-scale effects of microclimate on abundance and biting behaviours of two major malaria vectors, *An. arabiensis* and *An. funestus* in rural Tanzania.

## Methods

### Study area and period

**Figure 1.**
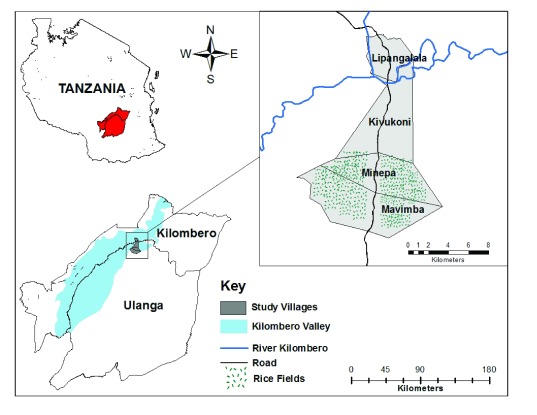
Map of the Kilombero and Ulanga districts showing the four study villages where entomological and environmental data were collected. (Kindly prepared by Doreen Siria)

Data on mosquito abundance and biting behaviour was collected from February 2015 to January 2016 in four villages covering two districts in the Kilombero river valley, south-eastern Tanzania ( Figure 1). The Kilombero valley ecosystem is dominated by a low-lying flood plain interspersed with villages and rice farms. There are two main seasons in the valley, a cool rainy season (February to June) and a hot dry season (July to October). There is also a short period of rains covers between November and January. The valley receives approximately 1200–1600 mm of rainfall annually and the mean daily relative humidity range from 54% to 71% while mean temperature ranges from 20°C to 32.6°C.

The dominant malaria vectors in the study area are *An. funestus* and *An. arabiensis*, but there are also other species such as *An. rivulorum,* that can carry malaria parasites, albeit in much lower rates ^52^. In combination, *An. funestus* and *An. arabiensis* populations in this area are predicted to generate an Entomological Inoculation Rate (EIR) of 18.45 infectious bites per person per year for unprotected individuals, with most of these infectious bites (86.2%) contributed by *An. funestus* ^52^. The main malaria intervention used in the area is LLINs ^53^.

### Mosquito sampling

Host seeking mosquitos were collected in four villages within the valley: Mavimba (8.3124°S, 36.6771°E), Minepa (8.2710°S, 36.6771°E), Kivukoni (8.2135°S, 36.6879°E) and Lipangalala (8.1539°S, 36.6870°E) ( Figure 1). Two houses were selected in each village, one in the middle of the village, and another towards the edge of the village. Working with trained, adult male volunteers, human landing catches (HLC) ^54^ were used for sampling mosquitoes hourly from 6pm to 6am for three or four consecutive nights each week (three nights/week in the wet season, four nights/week in the dry season), resulting in 12-16 sampling days per house per month, over a 12 month sampling period. On each night of sampling, one trained volunteer collected mosquitoes inside the house, and another collected within a 4-5m zone outside the house. Collected mosquitoes were put into separate cups, labelled by collection night and location. The volunteers rotated between indoor and outdoor positions after every hour to account for any biases due to variability in attractiveness of individuals to mosquitoes ^55, 56^. All *Anopheles* mosquitoes were identified to species group ( *An. funestus* s.l vs. *An. gambiae* s.l) based on morphology, and also their physiological status, as defined by being recently blood fed, unfed (without blood-meal) or gravid, was also recorded ^42^. A sub-sample of *An. funestus* s.l and *An. gambiae* s.l were subjected to molecular analysis at Ifakara Health Institute (details below).

### Environmental variables

Data on temperature and relative humidity were simultaneously recorded inside and outside of the houses, where mosquito collections were being conducted. The mean nightly temperature and humidity at each household was estimated from hourly values collected over the 12-hour sampling period (6pm to 6am) using Tinytag ^®^ data loggers (Gemini, UK). One logger was positioned inside in the middle of the room where sampling was conducted and the other was located outside near to the outdoor sampling point. Data on the total daily rainfall for the Kilombero valley was obtained from an electronic weather station maintained by Ifakara Health Institute (IHI), just outside Ifakara town, approximately 5km from the northernmost study village of Lipangalala.

### Laboratory analysis

A sub-sample of 2910 (25% of the total) female *An. gambiae* s.l. and 463 (61% of the total) female *An. funestus* s.l. mosquitoes were analysed using multiplex polymerase chain reaction (PCR) to identify their species ^57, 58^. Enzyme Linked Immunosorbent Assays (ELISA) were used to test for presence of *Plasmodium* parasites ^59^. The ELISA were done in pools of 10 mosquitoes or less. To prevent false positive results, the ELISA lysate was boiled for 10 minutes at 100°C, so as to eliminate heat-labile non *P. falciparum* protozoan antigens, which may constitute false positives in standard ELISA assays ^60^.

### Ethical statement

Ethical approval was obtained from the Ifakara Health Institute’s Institutional Review Board (IHI/IRB/No: 06-2016), and the Medical Research Coordination Committee of the National Institute for Medical Research in Tanzania (MRCC) (NIMR/HQ/R.8a/Vol.IX/2218). Approval for publishing this manuscript was obtained from the National Institute for Medical Research (NIMR), Ref: NIMR/HQ/P.12 Vol.XXII/30. Printed copies and web links to the publication will later be provided to NIMR after publication. Written informed consent was obtained prior to the start of each data collection from all volunteer mosquito collectors and household owners who agreed to participate in the study. Malaria tests were provided to all volunteers before, during and after the study, with the intention that only malaria-free individuals would be allowed to participate. All volunteers involved in HLCs were provided with prophylaxis (250 mg Mefloquine taken orally) once every week to prevent malaria infections during the course of the experiments. In addition, treatment (Coartem ^®^, 80 mg artemether and 480 mg lumefantrine for 3 days) was made available in case any of the participants became ill. However, none of the volunteers contracted malaria during the study.

### Statistical analysis of intra-annual (seasonal) patterns

Statistical analyses were conducted using R software version 3.3.2 ^61^. Generalized Additive Mixed Models (GAMM) was constructed to test the association between the nightly abundance of each vector species group (total number of female mosquitoes captured per person per night) and a set of environmental variables. The GAMM was fitted using the *gamm4* function implemented within the *mgcv* package ^62^. The use of GAMM is recommended in cases when the data (here mosquito counts) are not expected to have a linear relationship with some predictor variables (in our case, calendar days). This was certainly the case in our study where mosquitoes were trapped over a year-long period, during which their populations underwent large seasonal expansions and declines.

Initial models used Poisson likelihood, but over-dispersion (overdispersion statistic>2.0) necessitated the use of a negative binomial likelihood for modelling the abundance of *An. arabiensis* and *An. funestus*. The explanatory variables were: mean nightly temperature, mean nightly humidity, total daily rainfall, cumulative rainfall over a series of time lags, and sampling location (indoors or outdoors). The impact of both concurrent and time-lagged cumulative rainfall was investigated because both are known to have important, distinct impacts on mosquito abundance. For example, the amount of rain falling on the day of sampling may influence the “trap-ability” of mosquitoes, as they may refrain from flying during heavy rain ^63^. In addition, the size of the adult mosquito population is determined by the number of individuals emerging from aquatic larval habitats. As it takes ~2-3 weeks for mosquitoes to complete larval development in aquatic habitats, the cumulative amount of rain following in the weeks before sampling are probably a good indicator of the size of the adult population ^63^. Cumulative rainfall values over different time lags before each sampling day were calculated and used as separate explanatory variables to identify which time period was most informative of adult density. Rainfall variables used in the GAMM model included both rainfall on the day of sampling (0), and amount of rainfall accumulating 0, 1-2, 1-3, 1-4, 2-3, 2-4 and 3-4 weeks before each sampling day. These variables share common information, so cross correlations were a concern. To detect multi-collinearity, we used Variance Inflation Factors (VIF) to select a combination of uncorrelated covariates. Variables with VIFs>3 were not included in the model. Consequently, only cumulative rainfall at 0, 1-2, 2-3, and 3-4 weeks before the sampling day were included in the model together with other microclimatic variables measured on the sampling day.

Since mosquito catches are expected to be partially density dependent ^64, 65^, an auto-covariate was also included in the model as the number of mosquito collected two weeks before the sampling day. Days of the calendar year were included in the model as a smooth spline term to test whether there was a significant effect of season, with random effects included for household of collection, nested within village. The random effects aimed to capture unexplained variation that is consistent within households and to account for pseudo-replication within household and village. All the independent variables were centred and re-scaled to improve mixed model convergence.

A maximal model, with all explanatory variables and possible interactions, was constructed and sequentially compared with models containing fewer terms. These model comparisons were done using the Akaike Information Criterion (AIC) following existing procedures ^62, 66^. Deviance Explained (ED) by each model was obtained from the null deviance of an intercept-only model and the residual deviance of the candidate model.

### Statistical analysis of the relationship between exophilic and climatic condition

A second model was constructed to test for associations between the relative difference in microclimatic conditions (temperature and humidity) between indoor and outdoor sampling points and the degree of outdoor biting (exophily) in each *Anopheles* vector species. Generalized linear mixed models (GLMMs) fitted with a binomial likelihood for proportional data in *lme4* package were used ^67^. Exophily was calculated as number of mosquitoes caught outdoor ( *O*) as a proportion of the sum of the total caught indoors ( *I*) and outdoors ( *O*) between 6pm and 6am i.e. ( *O*_6 *pm*–6 *am*_)/( *I*_6 *pm*–6 *am*_ + *O*_6 *pm*–6 *am*_). Main predictor variables were the differences between indoor and outdoor temperature ( *ΔT*), relative humidity ( *ΔRH*), Indoor temperature and indoor humidity were also included as covariates in the model following exclusions of multicollinear candidate covariates by use of VIF. Model selection was done based on AIC (i.e. the lower the AIC value, the better the model) ^68^.

## Results

### Species composition and *Plasmodium* infection of *Anopheles* in study area

A total of 61,093 mosquitoes were collected inside and outside houses within the study area over the entire sampling period. Four mosquito genera were identified: *Culex* (72.74%), *Anopheles* (20.94%), *Mansonia* (5.94%) and *Aedes* (0.38%). A total of 12,795 *Anopheles* were collected, of which the major species group was *An. gambiae s.l.* (92.05%, Table 1) followed by *An. funestus* (5.98%), *An. pharoensis* (1.27%) and *An. coustani* (0.70%). Overall, 66.3% of *Anopheles* species were collected outdoors and 33.7% indoors ( Table 1). Most of the *Anopheles* species were captured in Minepa (71.4%, n=9,131, Table 1), followed by Kivukoni (13.8%, n=1,766), Mavimba (11.0%, n=1,403) and Lipangalala village (3.7%, n=495). Of the *An. gambiae s.l.* samples tested by PCR, the majority were confirmed as *An. arabiensis* (99.9%), and only one mosquito was found to be *An. quadriannulatus* (0.1%). The *An. funestus* group consisted of 77.2% *An. funestus s.s,* 20.3% *An. rivulorum* and 2.5% *An. leesoni*. The overall *Anopheles* PCR amplification rate was 83.2%. From all samples subjected to ELISA testing for malaria infection, only 5 (1.1%) sporozoite-positive individuals were detected in the *An. funestus* group, and none in *An. arabiensis.*

**Table 1.** Total number of *Anopheles* mosquitoes collected between February 2015 and January 2016 within the four localities.

Species	Village									
	Kivukoni		Lipangalala		Mavimba		Minepa		Total Overall	%
	Indoor	Outdoor	Indoor	Outdoor	Indoor	Outdoor	Indoor	Outdoor		
Anopheles arabiensis. *^+^	555	1,015	87	331	397	850	2,734	5,810	11,779	92.0
Anopheles funestus *	48	53	8	25	51	80	353	147	765	6.0
*Anopheles pharoensis*	14	52	0	5	4	10	20	57	162	1.3
*Anopheles coustani*	10	19	17	22	6	5	2	8	89	0.7
**Total**	**627**	**1,139**	**112**	**383**	**458**	**945**	**3,109**	**6,022**	**12,795**	**100.0**

* ^+^Only 1 specimen from the *An. gambiae s.l.* was identified as a species other than *An. arabiensis* (in this case, it was *Anopheles quadriannulatus)*. All the *An. gambiae s.l.* are therefore assumed to be *An. arabiensis* in this article. *Included in the final analysis.

**Figure 2.**
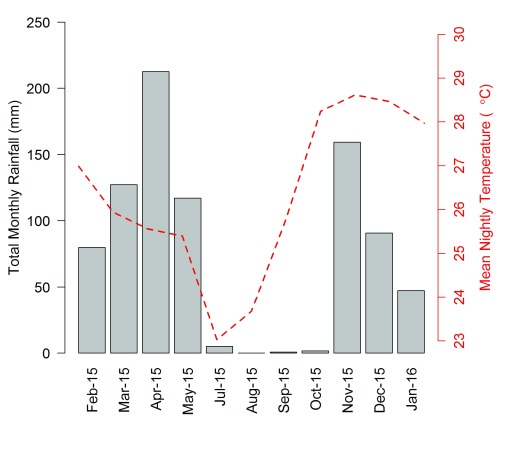
Total monthly rainfall (grey bars) and mean nightly temperature (red dashed-line) pattern in Kilombero valley.

During the study period, heavy rainfall occurred between March and May ( Figure 2), with precipitation ceasing in August, followed by a very dry 3-month period (~<5mm rainfall/week, August-October 2015). Mean temperatures were highest (> 28°C) in November and December (average rainfall of 27.6mm/week), and lowest (< 24°C) in July and August of 2015 ( Figure 2). On average, the microclimate inside houses was warmer and more humid than outdoors ( Table 2).

**Table 2.** Mean and range of temperature and relative humidity for both indoor and outdoor locations.

		Indoor	Outdoor
Temperature (°C)	Mean	26.30	26.00
	Range	19.35 – 31.55	19.83 – 30.65
RH. Humidity (%)	Mean	62.70	62.18
	Range	34.14 – 98.36	0 – 100

### Effects of microclimatic conditions on *Anopheles* species abundance

**Figure 3.**
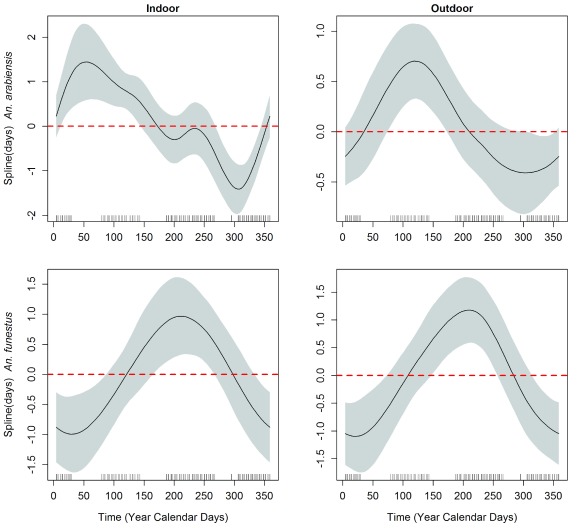
Anopheles vector abundance over time: Four panels showing cyclic cubic splines of seasonal variation in abundance as predicted by a Generalized Additive Mixed Model (GAMM) model.

GAMM models fitted with a negative binomial distribution provided a better representation of *An. arabiensis* and *An. funestus* abundance than those fitted with a Poisson distribution. For *An. funestus* the final model explained 39% of the null deviance. The smooth term (calendar days) indicated there was significant variation in abundance of *An. arabiensis* within a year both indoors (F = 42.31, effective degree of freedom (edf) =5.3, Figure 3) and outdoors (F = 16.68, edf=2.5, Figure 3). There was also significant variation in the abundance of *An. funestus* over the year both indoors (F = 12.26, edf=2.5, Figure 3) and outdoors (F = 18.48, edf=2.9, Figure 3). Preliminary analysis showed that the abundance of *An. arabiensis* varied significantly between indoor and outdoor locations, with approximately two times more being collected outdoors than inside after controlling for environmental variables ( Table 3). In contrast *An. funestus* abundance was similar between indoor and outdoor sampling locations ( Table 1 and Figure 5).

**Table 3.** Mean number of malaria vector species collected per person/night and absolute relative risks estimated GLMMs.

Species	Arithmetic Mean (b/p/n)		Absolute Relative Risk (95% CI)	
	Indoor	Outdoor	Indoor	Outdoor
*Anopheles* *arabiensis*	12.25	25.99	2.80 (0.58–13.51)	6.45 (1.34–31.08)
*Anopheles* *funestus*	1.49	0.99	0.30 (0.12–0.80)	0.32 (0.12–0.84)

Parameter estimates derived from the best models of mosquito abundance indicated that rainfall on the sampling day was positively associated with vector abundance. The abundance of *An. arabiensis* increased by ~16% while that of *An. funestus* increased by 26% for every 1mm increase in the amount of rain falling during the sampling day ( Table 4). The final model for *An. arabiensis* did not include the 1-2 weeks aggregated rainfall. Aggregated rainfall (2-3 weeks before sampling) was also positively related to *An. arabiensis* abundance ( Table 4). Aggregated rainfall (3-4 weeks before sampling) was also positively related to *An. arabiensis* and *An. funestus* abundance, with 31% and 43% increases in density predicted respectively for every 1mm increase in cumulative rain over this period. The final model for *An. funestus* did not include the 2-3 weeks aggregated rainfall. *Anopheles funestus* abundance was negatively associated with aggregated rainfall 1-2 weeks before sampling day.

**Table 4.** Results from the final GAMM model describing the predicted impacts of the climatic variables on the nightly abundance of *Anopheles* species.

Response variable	Independent Variable	Relative Risk * (95%CI)	ΔDev	p-value
*Anopheles arabiensis*	Mean night temperature	1.11 (0.92 – 1.34)	18.4	0.272
	Mean night humidity	0.58 (0.37 – 0.90)	103.2	**0.016**
	Rainfall on the capture day	1.16 (1.04 – 1.30)	78.5	**0.007**
	Aggregated rainfall 2 to 3 weeks	1.13 (1.00 – 1.28)	58.9	**0.049**
	Aggregated rainfall 3 to 4 weeks	1.31 (1.16 – 1.48)	141.2	**<0.001**
	Density dependence covariates	1.31 (1.10 – 1.56)	28.5	**0.003**
*Anopheles funestus*	Mean night temperature	1.66 (1.24 – 2.23)	2.95	**<0.001**
	Mean night humidity	1.55 (1.17 – 2.07)	1.6	**0.003**
	Rainfall on the capture day	1.26 (1.06 – 1.50)	24.7	**<0.001**
	Aggregated rainfall 1 to 2 weeks	0.81 (0.67 – 1.01)	1.3	0.059
	Aggregated rainfall 3 to 4 weeks	1.43 (1.18 – 1.74)	6.7	**<0.001**
	Density dependence variable	2.76 (1.88 – 4.03)	21.6	**<0.001**

*Relative Risk of greater than 1 indicated a positive association whereas less than 1 indicates a negative association.

Over the range of temperatures measured (19.4 – 31.6°C), an increase in the mean nightly temperature of 1°C was predicted to increase the overall abundance of *An. arabiensis* by ~11%, and that of *An. funestus* by ~66% ( Table 2). Lastly, an increase of one percentage point in the mean nightly humidity was associated with a reduction in the abundance of *An. arabiensis* by ~42% and increased *An. funestus* abundance by 55% ( Table 4).The mean daily abundance of both *An. arabiensis* and *An. funestus* was significantly associated with their density as measured two weeks prior to sampling, confirming temporal autocorrelation in population size. The density dependent terms was found to improve the model fitness and convergence of both *An. arabiensis* and *An. funestus*.

### Effects of temperature and relative humidity variation on the exophilic behaviour of *Anopheles* species

**Figure 4.**
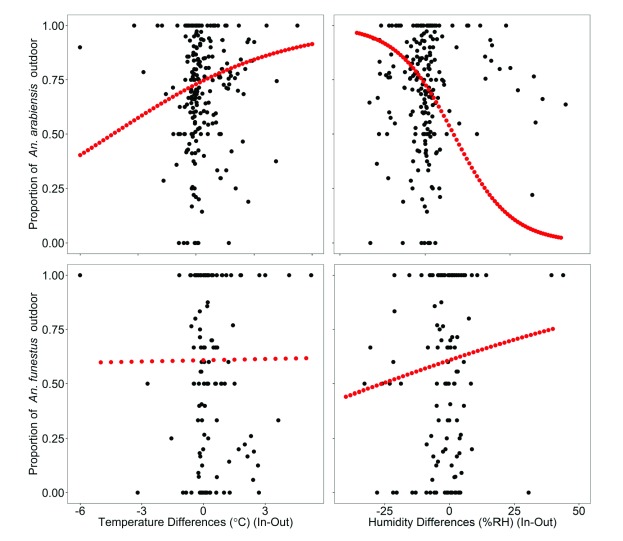
Relationships between microclimatic conditions and exophily behaviour of *Anopheles* mosquitoes. Black circles (observed) and red dotted (predicted values).

The GLMM with a binomial response variable (representing the proportion of mosquitoes caught outside) indicated that the relative difference in microclimatic conditions between indoor and outdoor environments had an impact on the degree of exophily in *An. arabiensis*. When temperatures were higher indoors compared to outdoors, the odds of exophily increased by ~26% in *An. arabiensis* for every one unit increase in temperature differential ( Table 5 and Figure 4). In contrast, for a one unit increase in the differential between indoor and outdoor humidity, the odds of exophily decreased by 6% ( Table 5 and Figure 4), within the limits of our microclimate measurements. There was an interaction between temperature differences ( *ΔT*) and humidity differences ( *ΔRH*). This interaction had the impact of increasing the degree to which exophily was enhanced by the indoor vs. outdoor temperature differential, when there was also a bigger difference in humidity between these habitats ( Table 5). In contrast, the proportion of *An. funestus* biting outdoors was not significantly related to temperature ( *ΔT*) or humidity ( *ΔRH*) difference between indoors and outside ( Table 5 and Figure 4).

**Table 5.** Results obtained from the final GLMM testing for associations of exophily (proportion of bites taken outdoors) and household-level microclimatic variables for two main *Anopheles* vector species.

Response variable	Independent Variable	Odds Ratio * (95%CI)	p-value
*Anopheles arabiensis*	Mean temperature difference ( *δT*)	1.25 (1.14 – 1.39)	**<0.001**
	Mean humidity differences ( *δRH*)	0.94 (0.89 – 1.00)	0.057
	*δT* ∗ *δRH*	1.12 (1.02 – 1.22)	**0.016**
*Anopheles funestus*	Mean temperature difference ( *δT*)	1.01 (0.76 – 1.32)	0.944
	Mean humidity differences ( *δRH*)	1.02 (0.55 – 1.88)	0.160
	Mean indoor temperature	1.39 (0.91 – 2.00)	0.124
	Mean indoor humidity	0.89 (0.58 – 1.37)	0.593
	InTemp *InHumid	0.63 (0.38 – 1.03)	0.066

*Odds ratio of greater than 1 indicated a positive association whereas less than 1 indicates a negative association.

**Figure 5.**
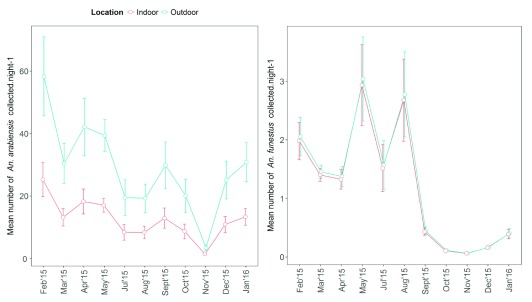
Seasonally predicted mean distribution of *Anopheles* mosquitoes captured per person per night pooled across study villages.

### Predictions for the seasonal abundance and biting behaviour of *Anopheles* mosquitoes

The best models for prediction of malaria vector abundance and biting behaviour as described above were used to investigate the degree to which human exposure to mosquito bites may be expected to vary seasonally in response to microclimatic conditions. Here, model predictions were obtained under a range of environmental conditions most typical of the wet and dry seasons. On the basis of these assumed typical values, the indoor biting rates of *An. arabiensis* were predicted to change from ~25 bites per person per night (b/p/n) during a typical wet season (March-May) to ~2 b/p/n during the dry season (August-October). While that of *An. funestus* would shift from ~1.5 b/p/n to below 0.5 b/p/n. The degree to which mosquito vectors attempt to feed outdoors is significantly dependent on indoor temperature relative to outdoor temperature, but this is not the case for *An. funestus*. Specifically, our model (GLMM) predicted that the proportion of *An. arabiensis* bites outside can shift from 72.9% when there is no temperature differences between locations, to a high up to 91.5% when indoor mean temperature is higher by 6ºC (maximum difference observed) ( Figure 4). We have also observed that, the exophily of *An. funestus* and *An. arabiensis* did not vary seasonally (wet vs. dry seasons) ( Figure 5).

## Discussion

We investigated associations between daily microclimatic variation in and around households, and also the abundance and biting behaviour of two major African malaria vectors over one year period in south-eastern Tanzania. Whilst previous studies have investigated seasonally-varying environmental drivers of vector abundance, few have explicitly investigated the role of microclimatic variation on preference of mosquitoes biting outdoors rather than inside of houses. Consistent with previous work ^63^, the present study detected strong seasonality in malaria vector abundance. Cumulative total rainfall occurring in the two weeks before sampling was a significant positive determinant of the densities of both *An. arabiensis* and *An. funestus*. The aggregated rainfall occurring 2–3 weeks, and 3–4 weeks before sampling day was positively related to *An. arabiensis* abundance, the latter having high impact on the abundance.

This 2 to 4 week lag period between rainfall and increased mosquito abundance is likely reflective of the period of time required by mosquitoes to lay their eggs (triggered by rainfall), have eggs hatch and complete larval development (1–2 weeks), then emerge as host seeking adult females (~4–5 days). A similar pattern has been observed in another study conducted in Kenya ^30^, which showed that rainfall lags of two weeks before sampling day, were positively correlated with abundance of *Anopheles* mosquitoes. One exception to the general finding of a positive effect of rainfall on vector abundance was the detection of a moderate, negative association between the amount of rainfall occurring 1-2 weeks before each sampling day and *An. funestus* nightly abundance. This finding contrasted with a positive association between *An. funestus* and cumulative rainfall over a longer time lag (e.g. 3-4 weeks before sampling). These differences in the effect of rainfall between vector species likely reflect differences in their larval ecology. Unlike *An. arabiensis* which often breeds in small, ephemeral aquatic habitats ^42^, *An. funestus* larvae can be found in larger, more permanent water bodies ^42, 69^.

The presence of large swamp areas in addition to other large ponds within the study area likely provide a stable year-round breeding site for *An. funestus*, which can be expected to decouple their dependency on seasonal rainfall ^29, 34^. Our GAMMs model predicted that both *An. funestus* and *An. arabiensis* could still be detected even after 2 to 3 months of very little/no rainfall, and that *An. funestus* densities peaked late into August ( Figure 2 and Figure 5). High rainfall during the sampling night tends to flush away immature mosquitoes from breeding habitat and also reduces catch-ability, though studies still consider high rainfall as ideal conditions for malaria transmission ^27, 63, 70^. Thus, the relationship between rainfall and vector population dynamics may be more complex than usually thought. Careful considerations of the interplay between longer-term and short-term effects are required to more accurately predict vector abundance.

In our study, the mean nightly abundance of both *An. arabiensis* and *An. funestus* was predicted to increase with temperature across the range of those measured indoors and outdoors. For every 1°C increase in temperature, *An. arabiensis* abundance was predicted to increase by about 11% and *An. funestus* by about 66%. This relationship should be used with caution because there is maximum temperature threshold at which *Anopheles* mosquitoes can survive ^24^. The minimum and maximum temperatures recorded in this study were 19.4°C and 31.6°C respectively. This range falls just below the maximum threshold of 32°C reported for *Anopheles* survival ^24^ and above the minimum temperature threshold of 18°C required for larval development ^37^. Previous studies have shown that a marginal increases in temperature above the minimum threshold (18°C) are associated with high mosquito densities, biting rates and the development of malaria parasites within mosquitoes ^27, 71, 72^. Such associations between mosquito and parasite life history and temperature are not expected to be linear, with temperatures above 32°C reported to reduce survival of some African *Anopheles* mortality ^24, 27^. When the temperature rises above this threshold, mosquito digestion rates also increase which later increases vector-host contact ^38, 39^. A study conducted in western Kenya on the duration of gonotrophic cycles using wild mosquitoes found that, an increase in average temperature reduces the first and second gonotrophic cycle length ^38^. Therefore, female *Anopheles* will need multiple blood meals to complete ovary development, hence high biting rates.

This study also generated some new insights on the potential for microclimatic variation to impact mosquito behaviour as well as their abundance. Previous laboratory work has shown that malaria vectors are able to sense temperature, and modify their choice of resting habitats in a pattern consistent with optimizing their survival ^13, 49, 73^. However, the role of temperature and other microclimatic conditions in determining the time and place of mosquito biting is less well understood. Though malaria vector species are known to have specific, distinct patterns of exophily ^14, 74, 75^, we hypothesized that there is some degree of flexibility within species to modify whether they bite in or outside of houses in response to fine-scale microclimatic variations. This was confirmed here by our finding that the proportion of outdoor biting by *An. arabiensis* is associated with relative difference in temperature and humidity between indoor and outdoor environments. Specifically, *An. arabiensis* were more likely to bite outdoors when conditions indoors were hotter and drier compared to outside. In contrast, the *An. funestus* remained generally endophilic (60.1%) regardless of fine-scale variation in temperature, humidity, and the relative difference in microclimate between indoor and outdoor settings. This indicates that at least this one major African vector species, *An. arabiensis*, tends to move toward cooler and more humid places, which are important in maintaining their survival.

Under controlled laboratory conditions *An. arabiensis* and *An. gambiae s.s.* are capable of detecting and responding to an increase in temperature of a few degrees by moving away from heat sources ^13, 76^. Mosquitoes use thermohygroreceptor cells to detect temperature changes ^47^, which is likely the primary mechanism through which they can assess conditions and modify their behaviour. Our findings reveal that *An. arabiensis* prefers biting in relatively cooler, humid places. This matches with laboratory observations where *An. gambiae s.l.*, *An. stephensi,* and *Cx. pipiens* moved toward the more humid and cooler parts of a cage (the roof), in comparison to other parts ^47^.

## Conclusions

Here we have shown that household-level microclimatic conditions strongly influence both the abundance and relative preference of malaria vectors for biting inside versus outside houses. Whilst previous work has also uncovered strong effects of temperature, humidity and, our study is unique in demonstrating an additional impact of microclimatic variation on vector biting behaviour. Exophily was related to the relative difference between indoors and outdoors temperature and in *An. arabiensis*, but not *An. funestus*. We have demonstrated that malaria vector *An. arabiensis* shifts the location of its biting from indoors to outdoors in association with relative differences in microclimatic conditions. Also, overall increments of household temperature as small as 1°C resulted in significant increases in the abundance of *An. funestus*. In order to improve on protection from LLINs, we will need to think more strategically not only about optimizing the type but timing of intervention deployment, to exploit vulnerabilities in their seasonal cycle of abundance and behaviour. These findings have implications for the fine-scale mapping of biting risk in households, and potential improvements in control measures by modulating household microclimates. This may also warrant consideration of seasonally targeted interventions as complementary strategies.

## Data availability

Data used to generate these findings are available from the Ifakara Health Institute data repository: http://dx.doi.org/10.17890/ihi.2016.01.99 ^77^.
