# Supplementary material for: Variations in household microclimate affect outdoor-biting behaviour of malaria vectors
Source: Wellcome Open Res. 2017 Oct 24;2:102. [Version 1] doi: 10.12688/wellcomeopenres.12928.1 (PMC5829465; doi:10.12688/wellcomeopenres.12928.1)
Supplement: Peer review history

# Peer review: Referee response for version 1

**DOI:** 10.21956/wellcomeopenres.14017.r28214

**Published:** 2017-12-18

**Authors:** Yaw A. Afrane

**Affiliations:** 1 Department of Medical Microbiology, College of Health Sciences, University of Ghana, Accra, Ghana

The authors investigated how variations in household microclimate affect outdoor-biting by malaria vectors, majorly *Anopheles arabiensis *and *Anopheles funestus*. This study is important to explain the recent increases in the tendency of otherwise endophilic and endophilic mosquitoes to rest and/or bite outdoors.

 The manuscript is sound in terms of the objectives and the findings. I do not have a lot of comments for the authors. However, the manuscript would be much clearer if the authors will dwell more on how the variations in the main microclimate parameters (temperature and humidity) affect mosquito densities outdoors and indoors. Rainfall in a particular day could affect the temperature and humidity of an area and therefore vector abundance indoors and outdoors.

I have read this submission. I believe that I have an appropriate level of expertise to confirm that it is of an acceptable scientific standard.

# Peer review: Referee response for version 1

**DOI:** 10.21956/wellcomeopenres.14017.r27286

**Published:** 2017-11-21

**Authors:** Joseph M Mwangangi

**Affiliations:** 1 Centre for Geographic Medicine Research-Coast (CGMR-C), Wellcome Trust Research Programme, Kenya Medical Research Institute (KEMRI), Kilifi, Kenya

The manuscript is well written. The study design and data analysis are well articulated and well written. The methodology is well written and captures all details of the study. In the study areas, it would be good to comment on the types of houses and how they are distributed/or clustered within the 4 villages. The results are comprehensive to answer the specific objectives in the study. Discussion is well written and covers all the key aspects of the study. The references cited in this paper are relevant and are sufficient. The conclusion is based on the results of this study. I would like to recommend the publication of this paper in your journal.

I have read this submission. I believe that I have an appropriate level of expertise to confirm that it is of an acceptable scientific standard.

# Peer review: Referee response for version 1

**DOI:** 10.21956/wellcomeopenres.14017.r27281

**Published:** 2017-11-20

**Authors:** Krijn Petrus Paaijmans

**Affiliations:** 1 Barcelona Institute for Global Health (ISGlobal), Barcelona, Spain

While climate x mosquito abundance interactions are well-understood, climate x mosquito behavior interactions are understudied. Yet understanding mosquito behaviors can be more informative (and thus important) to assess the gap in protection by interventions such as IRS and LLINs. Therefore I really appreciate this manuscript.

**Major comments**

**(1) Recording of microclimatic data**

 Considerable thinking has been devoted to the study design when it comes to mosquito collections (village selection, position of mosquito collectors over time, etc), but when it comes to the description of the collection of microclimatic data, no details are given (note that this is not unique to this paper). As the results/conclusions rely heavily on the collected temperature (T) and humidity (RH) data, there needs to be a detailed section in the methodology on data collection, including the accuracy of the loggers, calibration (these loggers normally tend to be used in different environments for years), rotation of loggers (between indoor and outdoor environment, between villages), placement height (similar indoors as outdoors? Small differences in height will affect T).

**(2) Microclimatic parameters**

 The microclimatic variables included in the analysis are (1) mean nightly T, (2) mean nightly RH, and (3) relative difference between indoor and outdoor T/RH (I assume this is based on the differences in the mean). I was wondering whether you examined (1) the difference between nightly minimum and maximum, (2) the slope of decrease over a certain time period during the night (e.g. to assess the impact of small vs large drops in T), or similar indicators to assess if small (micro-temporal) changes in the environment lead to a change in behavior. In addition, it might be worth looking at potential evapotranspiration (PE), as this is known to affect mosquitoes.

**(3) Number and type of houses selected**

 Indoor microclimate can differ considerably between different house types, over space and over time. You have selected only 2 houses per study area. How representative were these houses? What was the variation in microclimate between houses and the villages? This information is essential to understand the significance of the findings.

**(4) Additional analysis**

 Related to point 2: As you have hourly biting and hourly climatic data, which you could relate, is it possible to do a more detailed analysis on if/how microclimate impacts the time of biting?

**(5) Interpretation**

 There is a known sampling bias associated with HLC (related to the unnatural behavior of sitting outdoors throughout the night, while the majority of the local human population may actually be asleep indoors). As such – in nature – there will be a microclimate x host availability interaction (will they still bite outdoors under those climatic conditions if there are no/limited hosts?). This needs to be highlighted and discussed. How relevant are your data ‘in real life’?

**Minor comments**

 Figures: Include relevant temperature data (now only one line for temperature is presented in Figure 2)

I have read this submission. I believe that I have an appropriate level of expertise to confirm that it is of an acceptable scientific standard, however I have significant reservations, as outlined above.
